# Individuals with substance use disorders experience an increased urge to move to complex music

**DOI:** 10.1073/pnas.2502656122

**Published:** 2025-05-12

**Authors:** Jan Stupacher, Benedetta Matarrelli, Danilo Cozzoli, Mario Ventura, Francesco Montinaro, Luciana de Gennaro, Peter Vuust, Elvira Brattico

**Affiliations:** ^a^Center for Music in the Brain, Department of Clinical Medicine, Aarhus University and The Royal Academy of Music Aarhus/Aalborg, Aarhus 8000, Denmark; ^b^Department of Education, Psychology, Communication, University of Bari, Bari 70121, Italy; ^c^Department of Bioscience, Biotechnology and Environment, University of Bari, Bari 70121, Italy; ^d^Institute of Genomics, University of Tartu, Tartu 50090, Estonia

**Keywords:** groove, pleasurable urge to move to music, dopamine, cocaine, heroin

## Abstract

Substance use disorders disrupt the dopaminergic system of the human brain, which plays a central role in movement and reward processing, altering perception, and cognition. The pleasurable urge to move to music, known as groove, relies on dopamine for reward, anticipation, beat perception, and motor system activity. Using a well-established paradigm, which shows an inverted-U relationship between groove and musical complexity, we investigated how dopamine downregulation from long-term cocaine and heroin use affects the experience of music. Drug users experienced stronger groove with high rhythmic and harmonic complexities than nonusers, while moderate complexities elicited similar responses across groups. This pattern differs from other populations with altered dopaminergic function, such as Parkinson’s disease or musical anhedonia, highlighting a distinct effect of drug addiction on music perception. The findings suggest that drug users seek more intense and complex stimulation, supporting the hypothesis that a hypodopaminergic state associated with drug use raises the threshold for nondrug stimuli to engage the reward system.

Rewarding experiences involve a core brain network including dopaminergic nuclei (1). Repeated drug-induced dopamine surges, which exceed those elicited by natural rewards, reduce dopamine release and receptors in the striatum of individuals with substance use disorders (SUD), potentially raising the threshold for nondrug environmental stimuli to engage the dopamine system and elicit reward (2). Although most addiction theories propose a unified mechanism across different substances, psychostimulants and opioids—such as cocaine and heroin—may have distinct behavioral and neurobiological effects (3).

Dopamine not only shapes reward, motor, and arousal components underlying drug addiction; it also plays a central role in experiencing music, particularly the pleasurable urge to move to music known as *groove*. Several studies indicate that music with moderate, compared to low or high, rhythmic complexity induces the strongest groove experience (4–6). The resulting inverted-U relationship between rhythmic complexity and groove can be interpreted within the framework of predictive processing (7): Moderately complex rhythms generate the highest number of strongly weighted prediction errors, which occur when the internally generated model of beat and meter and the rhythmic input mismatch (Fig. 1). Resolving these prediction errors is proposed to increase motor system activity and reward, involving limbic- and motor-associated basal ganglia nuclei (4, 7, 8). Consistent with this perspective, the dorsal striatum in the nigrostriatal dopamine pathway is implicated in rhythm perception and beat anticipation (9), and the ventral striatum in the mesolimbic dopamine pathway is associated with music-induced pleasure (10). Dopamine depletion in Parkinson’s disease flattens the inverted-U of groove, potentially due to disruption of rhythm-related predictive processes (11). Additionally, stronger groove experiences may lead to an upregulation of norepinephrine (12), which is linked to dopamine signaling.

**Fig. 1.**
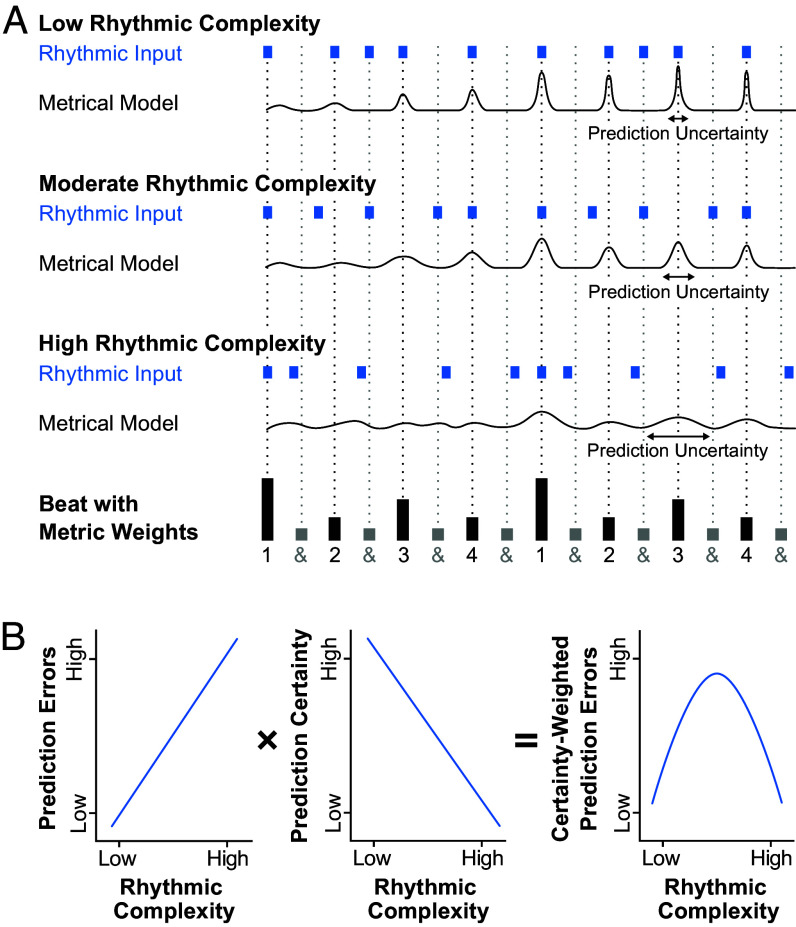
(*A*) Blue squares mark onsets of the three rhythms (see ref. 13). The *Bottom* row shows metric weights in 4/4 meter. Black traces represent exemplary mental models of beat and meter, outlined as probability distributions for illustration; distribution means reflect the time of predicted beats, widths indicate prediction certainties. (*B*) The inverted-U shape of certainty-weighted prediction errors (*Right*) as a combination of increasing number of prediction errors (*Left*) and decreasing prediction certainty (*Middle*) with increasing rhythmic complexity (see ref. 4).

To better understand how dopamine influences music perception, movement, and reward, we examined how SUD affect groove experiences. We compared ratings of the urge to move to music between male participants in rehabilitation for cocaine addiction (*n* = 19, mean age = 36.9), heroin/cocaine addiction (*n* = 16, mean age = 38.5), and a control group without drug addiction (*n* = 23, mean age = 30.9). Ratings were given in response to stimuli varying in rhythmic and harmonic complexity, previously used in groove studies (13) (Fig. 1). We expected groove experiences in SUD participants to either resemble the flattened responses observed in people with Parkinson’s (11), the comparable-to-control responses seen in musical anhedonics (14), or to align with the elevated reward threshold hypothesis (2).

## Results and Discussion

**Fig. 2.**
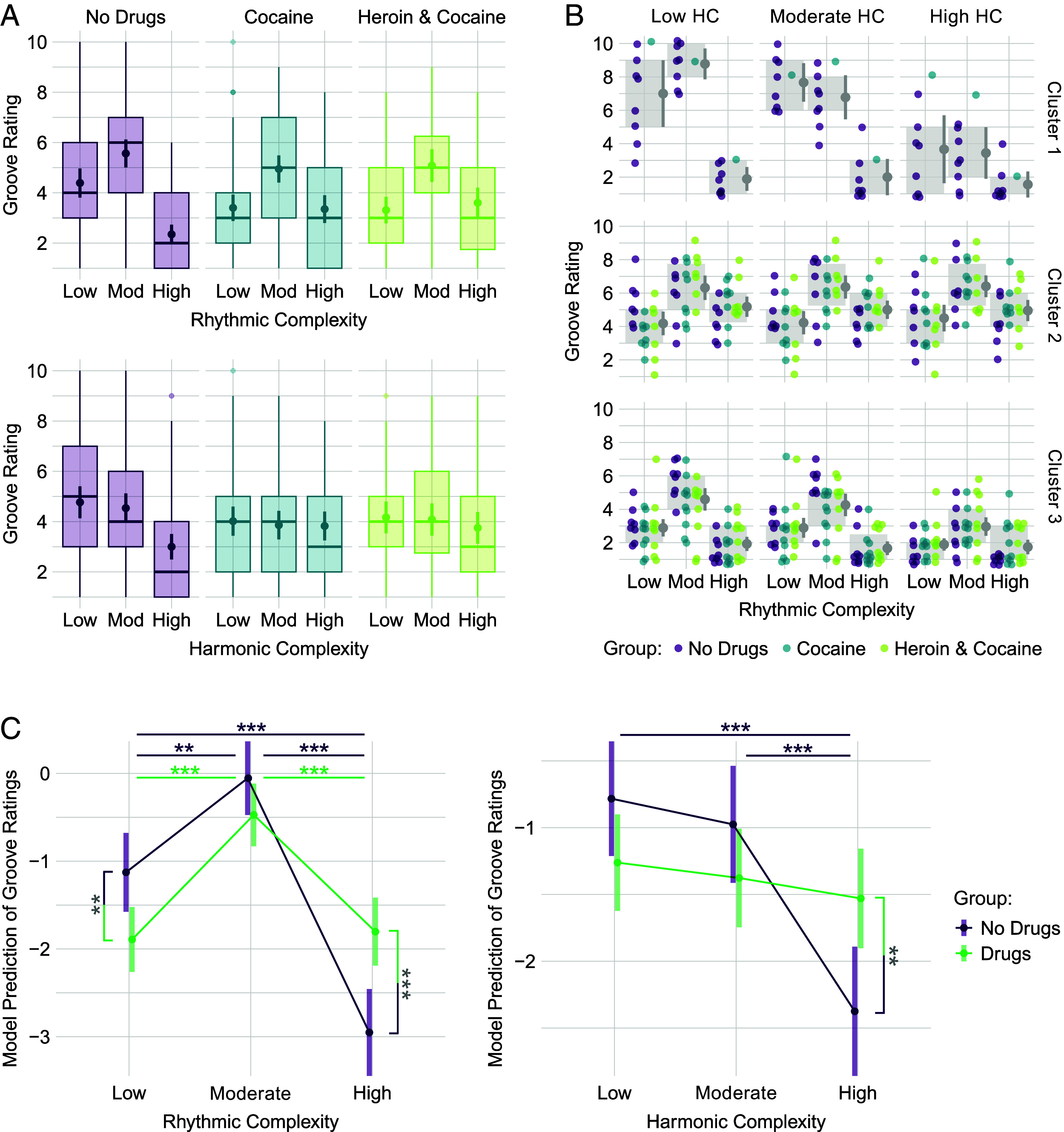
(*A*) Groove ratings of nonusers, cocaine users, and heroin/cocaine users across rhythmic (*Top*) and harmonic (*Bottom*) complexity levels. Dots represent means with 95% CI. Boxplots show medians, first and third quartiles, values within and outside 1.5 × interquartile range. (*B*) K-means clustering results. Each dot represents a participant’s groove rating. Gray boxes mark first and third quartiles and gray dots represent means with 95% CI. Nonusers are predominantly grouped in Cluster 1, which is characterized by low ratings for high complexities. Clusters 2 and 3 do not differentiate between cocaine and heroin/cocaine users and indicate that high complexity ratings are less negative compared to low and moderate complexities. (*C*) Predicted groove ratings from the cumulative link model. *RC* × *Drug Group* interaction: *χ^2^*(2) = 15.93, *P* < 0.001; *HC* × *Drug Group* interaction: *χ^2^*(2) = 6.98, *P* = 0.031. *RC* × *HC* and *RC* × *HC* × *Drug Group* interactions were nonsignificant (both *P* > 0.18). ***P < 0.001, **P < 0.01 in Tukey-corrected pairwise comparisons.

Rhythmic and harmonic complexity levels differently affected groove experiences in drug users and nonusers. Groove ratings did not considerably differ between cocaine and heroin/cocaine users (Fig. 2 *A* and *B*), suggesting a common effect of drug addiction-related dopamine depletion. We therefore compared a combined group of drug users (*n* = 35) with nonusers in a cumulative link model: *Groove Rating ~ Rhythmic Complexity × Harmonic Complexity × Drug Group*, with participants as random intercepts.

Rhythmic Complexity (RC): The influence of *RC* on the experience of groove differed between drug users and nonusers (Fig. 2*C*). Group comparisons showed that drug users experienced stronger groove with *high RC* (*z* = −3.9, *P* < 0.001) and weaker groove with *low RC* (*z* = 2.8, *P* = 0.005) than nonusers, whereas *moderate RC* did not significantly differ between groups (*z* = 1.5, *P* = 0.122). In nonusers, *moderate RC* was rated highest, followed by *low* and *high RC*, replicating previous studies (4, 13). In drug users, *moderate RC* was also rated highest, but in contrast to nonusers the difference between *low* and *high RC* was not significant. The stronger groove experiences with *higher RC* in drug users differ from findings in musical anhedonics, who rate the urge to move to music similar to controls (14). In addition, unlike people with Parkinson’s, who show a flattened inverted-U of groove ratings (11), drug users still show peak groove experiences with *moderate RC*.

Harmonic Complexity (HC): Drug users and nonusers were differently affected by *HC* (Fig. 2*C*). Group comparisons showed that drug users experienced stronger groove with *high HC* (*z* = −2.9, *P* = 0.003) than nonusers, whereas ratings of *low* (*z* = 1.8, *P* = 0.079) and *moderate HC* (*z* = 1.5, *P* = 0.147) did not significantly differ between groups. Nonusers experienced weaker groove with *high HC* compared to *low* and *moderate HC*, whereas *HC* levels did not significantly differ in drug users.

Collectively, the results indicate that drug users experience stronger groove with higher rhythmic and harmonic complexities than nonusers. This supports the hypothesis that drug addiction-related decreases in dopamine release and receptors in the striatum raise intensity thresholds for environmental stimuli to be perceived as engaging and rewarding (2). The drug users’ reduced engagement with low rhythmic complexity corroborates this interpretation. The results also align with sensation seeking—a trait characterized by the pursuit of novel and complex experiences—which is associated with increased preferences for intense music (15) and the development of drug use (16). Our findings suggest that individuals with SUD experience high-complexity music as more groove-inducing than nonusers because it provides intense enough stimulation to engage their downregulated dopaminergic networks.

This study offers a different perspective on the unique musical experiences of individuals with SUD and the potential involvement of the dopaminergic reward system. It establishes a foundation for future studies to incorporate neuroimaging, examine additional factors, such as general cognitive function or dance experience, and test the effects of addiction to other substances. Given the universal drive to drum, sing, and dance in groups (17), the phenomenon of groove presents valuable opportunities to study movement-reward links and develop music-supported interventions to enhance well-being and social connection in SUD.

## Materials and Methods

All SUD participants resided in rehabilitation centers where substance use was strictly prohibited (Therapeutic Community Emmanuel, Italy). Most cocaine users took cocaine for 10 or more years (n = 10) or 4 to 10 y (n = 6), and none had used heroin. Most heroin/cocaine users took heroin for 10 or more years (n = 7) or 4 to 10 y (n = 5) and cocaine for varying durations. Control participants reported no use of cocaine or heroin, except one who tried cocaine on a sporadic occasion. All participants who agreed to participate completed the study. Written informed consent was obtained in accordance with the Declaration of Helsinki. Ethics approval was obtained (Commissione Etica, Department of Education, Psychology, Communication, University of Bari, ET-23-11 and Comitato Etico Locale, IRCCS Oncologic Hospital Giovanni Paolo II, Bari, 2018-PDR-01136). Data, code, and materials: researchbox.org/3908 (18). Details in *SI Appendix*.

## Supplementary Material

Appendix 01 (PDF)

## Data Availability

Anonymized ratings and demographic data have been deposited in ResearchBox (https://researchbox.org/3908) (18).
