# Supplementary material for: Individuals with substance use disorders experience an increased urge to move to complex music
Source: Proc Natl Acad Sci U S A. 2025 May 12;122(20):e2502656122. doi: 10.1073/pnas.2502656122 (PMC12107138; doi:10.1073/pnas.2502656122)
Supplement: Supplementary file 1 — Appendix 01 (PDF) [file pnas.2502656122.sapp.pdf]

PNAS

Supporting Information for

Individuals with substance use disorders experience an increased urge to move to complex music

Jan Stupacher, Benedetta Matarrelli, Danilo Cozzoli, Mario Ventura, Francesco Montinaro, Luciana de Gennaro, Peter Vuust, Elvira Brattico

Corresponding authors: Jan Stupacher and Elvira Brattico
Email: stupacher@clin.au.dk, elvira.brattico@clin.au.dk

Methods

Participants. This study integrates participants from the project GrooveSUD focusing on the groove experience in substance use disorders (SUD) and a subset of participants who completed the groove rating task as part of the project BioSUD, which aimed to investigate the etiology of SUD in Italy. The GrooveSUD project was approved by the ethics review board “Commissione Etica” of the Department of Education, Psychology, Communication, University of Bari, Italy (ET-23-11), and the BioSUD project was approved by the ethics board “Comitato Etico Locale” of the IRCCS Oncologic Hospital of Bari Giovanni Paolo II (2018-PDR-01136). The study included an experimental group with SUD recruited from the Therapeutic Community Emmanuel in the three Italian towns of Lecce, Oria, and Triggiano, and a control group without SUD recruited during university lectures and through social media. Participants in the experimental group resided in rehabilitation centers where substance use was strictly prohibited. They consisted of cocaine users (n = 19 males, mean age = 36.9, SD = 10.1) and heroin/cocaine users (n = 16 males, mean age = 38.5, SD = 8.6). Cocaine users reported using cocaine for 10 or more years (n = 10), 4–10 years (n = 6), 3 years (n = 1), 2 years (n = 1), and 1–6 months (n = 1). None of them took heroin. Heroin/cocaine users reported heroin use for 10 or more years (n = 7), 4–10 years (n = 5), 3 years (n = 2), and 1 month (n = 2), with cocaine use ranging from 1 month to more than 10 years. Participants in the control group (n = 23 males, mean age = 30.9, SD = 9.1) reported no use of cocaine or heroin, except for one individual who used cocaine for less than a month. Comparable sample sizes were used in groove-rating studies by Romkey et al. (1) with musical anhedonics (n = 17 anhedonics, n = 17 controls) and Pando-Naude et al. (2) with Parkinson’s participants (n between 23 and 27). All participants received detailed written information about the study and provided written informed consent in accordance with the Declaration of Helsinki. For participants with SUD, additional meetings were conducted by EB and BM to present the procedures, aims, and scientific aspects of the study (without revealing any hypotheses) in spaces provided by the therapeutic communities. Since no reimbursement was provided, these presentation meetings were crucial for motivating and encouraging participation on a volunteer basis. Control participants were university students or personnel who, after being informed about the procedures, aims, and scientific aspects of the study—similar to SUD participants—volunteered to participate.

Materials. Each participant listened to nine musical stimuli characterized by low, moderate, and high rhythmic and harmonic complexity, as employed in previous studies (3,4). Rhythmic complexity was manipulated by levels of syncopation and harmonic complexity was varied between a major triad and two inversions (low complexity), four-note chords with extensions (moderate complexity), and adding a flat ninth interval between chord note and extension (high complexity). For further details see (3,4). Stimuli lasted 10 seconds and consisted of five piano chords at each complexity level. All stimuli are available online: researchbox.org/3908. After listening to each stimulus, participants answered the question, "How much does the music you listened to make you want to move?" on a ten-point scale from 1, indicating no desire to move, to 10, indicating a strong desire to move. After the ratings, participants filled out questionnaires about substance use behaviors, associated clinical symptoms, pharmacological treatments, and demographic data including age, gender, musical training (yes/no), education, and income.

Procedure. SUD participants from the Lecce community completed the task in a silent room at the "Vito Fazzi" Hospital. SUD participants from Triggiano and Oria communities completed the task in locations provided by health care operators of their therapeutic communities. Questionnaires were administered in small groups under the supervision of psychologists and community staff. Control participants completed the same tasks in designated spaces at the University of Bari. For all participants, groove ratings were always given individually in an acoustically isolated room, using the same computer and closed-back over-ear headphones (Roland RH-5). The instructions for the

groove rating task were identical for drug users and nonusers. The study's design and testing environment were standardized to minimize variability between the experimental and control groups.

Statistical Analysis. To identify underlying patterns in groove ratings, we applied k-means clustering, an unsupervised data-driven method that groups responses based on similarity. Given the three experimental groups (cocaine users, heroin/cocaine users, and nonusers), we selected a three-cluster solution using data from all nine combinations of harmonic and rhythmic complexities. As groove ratings did not considerably differ between cocaine and heroin/cocaine users, we fit the following cumulative link model for ordinal data using the *ordinal* package (5) in *R* (6): *Groove Rating ~ Rhythmic Complexity (RC) × Harmonic Complexity (HC) × Drug Group* (drug users vs nonusers), including participant as a random intercept. To assess the significance of the fixed effects, we performed type III analyses of deviance with Wald chi-square tests. Pairwise comparisons were computed with the *emmeans* package (7) for R. To test the influence of age, musical training, and socioeconomic status, we compared the models

- *Groove Rating ~ RC × HC × Drug Group × Age*
- *Groove Rating ~ RC × HC × Drug Group × Musical Training (yes/no)*
- *Groove Rating ~ RC × HC × Drug Group × Socioeconomic Status*

to the original model (*Groove Rating ~ RC × HC × Drug Group*). Information on musical training was only available for 15 drug users and 20 nonusers. Four of 15 drug users and 10 of 20 nonusers had musical training. Socioeconomic status was operationalized as the mean of two indicators: the highest level of education (1 = elementary school, 2 = middle school, 3 = high school, 4 = master's degree, 5 = postgraduate) and annual income in Euro (1 = less than 8,000, 2 = 9,000-17,000, 3 = 17,000-26,000, 4 = 27,000-40,000, 5 = more than 41,000). Drug users had a significantly lower socioeconomic status (2.17, $SD = 0.74$) than nonusers (3.89, $SD = 0.67$; $p < .001$ in a Wilcoxon rank sum test). Importantly, neither age, musical training, nor socioeconomic status significantly improved the model fit ($p = .155$, $p = .753$, and $p = .872$, respectively).

SI References

1. I. D. Romkey, T. Matthews, N. Foster, S. Dalla Bella, V. B. Penhune, The pleasurable urge to move to music is unchanged in people with musical anhedonia. *Plos One* **20**, e0312030 (2025).
2. V. Pando-Naude, et al., Dopamine dysregulation in Parkinson’s disease flattens the pleasurable urge to move to musical rhythms. *European Journal of Neuroscience* **59**, 101–118 (2024).
3. J. Stupacher, M. Wrede, P. Vuust, A brief and efficient stimulus set to create the inverted U-shaped relationship between rhythmic complexity and the sensation of groove. *Plos One* **17**, e0266902 (2022).
4. T. E. Matthews, M. A. G. Witek, O. A. Heggli, V. B. Penhune, P. Vuust, The sensation of groove is affected by the interaction of rhythmic and harmonic complexity. *Plos One* **14**, e0204539 (2019).
5. R. Christensen, O*rdinal – Regression Models for Ordinal Data*. https://CRAN.R-project.org/package=ordinal (2023).
6. R Core Team, *R: A language and environment for statistical computing*. R Foundation for Statistical Computing, Vienna, Austria. https://www.R-project.org/ (2021).
7. R. V. Lenth et al., *Package ‘emmeans’*. https://CRAN.R-project.org/package=emmeans (2023).
